# Distributed Joint Optimization of Beamforming and Power Allocation for Maximizing the Energy Efficiency of Cognitive Heterogeneous Networks

**DOI:** 10.3390/s21093186

**Published:** 2021-05-04

**Authors:** Kisong Lee

**Affiliations:** Department of Information and Communication Engineering, Dongguk University, Seoul 04620, Korea; kisonglee@dongguk.edu; Tel.: +82-2-2260-3233

**Keywords:** cognitive heterogeneous networks, MISO interference channel, energy efficiency, joint optimization, distributed algorithm

## Abstract

This paper investigated an energy-efficient beamforming and power allocation strategy for cognitive heterogeneous networks with multiple-input-single-output interference channels. To maximize the sum energy efficiency of secondary users (SUs) while keeping the interference to primary networks under a predetermined threshold, I propose a distributed resource allocation algorithm using dual methods, in which each SU updates its beamforming vector and transmit power iteratively without any information sharing until convergence. The simulation results verify that the performance of the proposed scheme is comparable to that of the optimal scheme but with a much shorter computation time.

## 1. Introduction

With the rapid increase in mobile traffic and wireless devices, cognitive heterogeneous networks (CHNs) have received a great deal of attention as a promising infrastructure for improving data rates and communication coverage [1,2]. The spectral efficiency can be significantly improved by sharing the same spectrum between different types of networks in CHNs, which is not possible with conventional homogeneous networks; however, at the same time, severe co-channel interference can occur. Accordingly, a number of studies have been conducted regarding developing methods for mitigating co-channel interference between different networks, such as interference coordination and cancellation [3,4,5], radio resource management [6,7], and cooperative strategy [8,9].

The explosive growth in mobile traffic has also led to a considerable increase in the energy consumption of wireless devices. Given that most wireless devices are powered by limited batteries, energy shortages are regarded as one of the main obstacles for limiting the performance of CHNs [10].

In this context, there have been attempts to develop energy-efficient communications for CHNs [11,12,13,14]. In [11,12], resource allocations were investigated to maximize the energy efficiency of underlying device-to-device (D2D) communications, and, in [13,14], energy-efficient user-association methods were also investigated. To further improve the energy efficiency of CHNs, studies on multi-antenna techniques have been undertaken [15,16,17,18]. In [15], joint beamforming and power allocation were designed to maximize the energy efficiency of a point-to-point multiple-input-single-output (MISO) distributed antenna system. An energy-efficient framework was provided for CHNs with multiple-input-multiple-output (MIMO) in [16], and a beamforming design for energy-efficient CHNs was proposed in consideration of co-channel interference in [17]. A joint power allocation and reflecting beamforming was also proposed based on reinforcement learning to enhance anti-jamming communication performance and mitigate jamming interference in [18].

Although some existing works have investigated strategies for energy-efficient CHNs, they used a centralized approach to deal with the non-convex optimization problems [11,12,13,17]. Given that this centralized approach needs high computational complexity and large signaling overhead, it is required to devise a distributed algorithm for energy-efficient CHNs that can be operated in practical systems.

This paper considered CHNs with MISO interference channels, in which the secondary user (SU) pairs opportunistically to utilize the same spectrum as long as the interference on the primary networks does not exceed the allowed threshold. I formulated a problem that optimizes the beamforming vectors and transmit powers of SU pairs jointly to maximize the sum energy efficiency while ensuring the constraint of the interference to primary networks.

An energy-efficient beamforming and power allocation strategy using dual methods, which can be operated in a distributed manner without any information sharing is proposed to deal with the formulated non-convex problem. Evaluating the performances of the proposed scheme under a variety of environments shows that the proposed scheme achieved near-optimal sum energy efficiency and violation probability while reducing the computation time significantly.

The remainder of this paper is organized as follows. In Section 2, the considered system model is introduced, together with the problem statement, and the energy-efficient beamforming and power allocation is proposed in Section 3. In Section 4, the performance of the proposed scheme is evaluated under a variety of scenarios, and finally the conclusions are presented in Section 5.

## 2. System Model and Problem Statement

**Figure 1:**
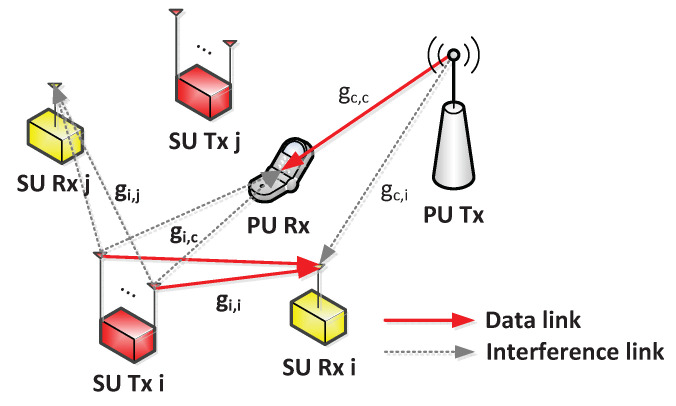
System model for cognitive heterogeneous networks with MISO interference channels.

As shown in Figure 1, I considered CHNs with MISO interference channels, in which *N* SU pairs, each of which is composed of a transmitter (Tx) equipped with *K* antennas and a receiver (Rx) equipped with a single antenna [15,19], share the same spectrum with primary users (PUs) equipped with a single antenna. The sets of SU pairs and antennas are denoted as N and K, respectively, i.e., |N|=N and |K|=K. The channel between SU Tx *i* and SU Rx *j* for antenna *k* is also denoted as $g_{i,j}^{\left[k\right]}$, and the index *c* for PUs is used, e.g., $g_{i,c}^{\left[k\right]}$ is the channel between SU Tx *i* and PU Rx for antenna *k*, and $g_{c,i}$ is the channel between the PU Tx and SU Rx *i*. Each SU Tx can transmit a data signal to its paired SU Rx through the same spectrum band as long as it does not interfere with the data transmission of PUs. $g_{i,c}^{\left[k\right]}$ for k∈K is known at SU Tx *i* to regulate the amount of interference on the PU Rx below a predefined threshold.

The received signal at SU Rx *i* is represented by

(1) $$\begin{array}{c} y_{i}=\sqrt{p_{i}}g_{i,i}^{H}w_{i}x_{i}+\sum_{j\in N\backslash \{i\}}\sqrt{p_{j}}g_{j,i}^{H}w_{j}x_{j}+\sqrt{p_{c}}g_{c,i}x_{c}+z_{i}, \end{array}$$

where ${\left(\cdot \right)}^{H}$ denotes a Hermitian transposition, $x_{i}$ and $x_{c}$ are the normalized data symbols sent by SU Tx *i* with transmit power $p_{i}$ and the PU Tx with transmit power $p_{c}$, respectively, and $z_{i}∼\mathrm{CN}\left(0,\sigma^{2}\right)$ indicates additive white Gaussian noise at SU Rx *i*. In addition, $g_{i,j}=\left\{g_{i,j}^{\left[1\right]},g_{i,j}^{\left[2\right]},\cdots ,g_{i,j}^{\left[K\right]}\right\}\in C^{K\times 1}$, and $w_{i}$ is a beamforming vector with a unit norm, such that $w_{i}=\left\{w_{i}^{\left[1\right]},w_{i}^{\left[2\right]},\cdots ,w_{i}^{\left[K\right]}\right\}\in C^{K\times 1}$ and $∥w_{i}{∥}^{2}=1$.

The achievable spectral efficiency of the SU pair *i* is given by

(2) $$\begin{array}{cc} r_{i} & =\log_{2}\left(1+\frac{p_{i}{\left|g_{i,i}^{H}w_{i}\right|}^{2}}{\sigma^{2}+p_{c}|g_{c,i}{|}^{2}+\sum_{j\in N\backslash \{i\}}p_{j}{\left|g_{j,i}^{H}w_{j}\right|}^{2}}\right). \end{array}$$

The consumed power at the SU pair *i* is expressed as

(3) $$\begin{array}{cc} P_{i}^{CE} & =P_{\mathrm{circuit}}+p_{i}, \end{array}$$

where $P_{\mathrm{circuit}}$ is the constant power consumed in communication circuits. From (2) and (3), the energy efficiency of the SU pair *i* can be defined as the spectral efficiency divided by the power dissipation (bits/Hz/Joule), i.e., $\eta_{i}^{EE}=\frac{r_{i}}{P_{i}^{CE}}$, which implies how efficiently energy is used for data transmission. At the same time, the transmission from SU Tx *i* causes interference to the PU Rx; this interference is expressed as $I_{i}=p_{i}{\left|g_{i,c}^{H}w_{i}\right|}^{2}$.

Then, the problem is developed to find the optimal resource allocation strategy of SU pairs, i.e., the beamforming vectors and transmit powers, to maximize the sum energy efficiency while ensuring that the interference on the PU Rx is less than the maximum allowable level, $I_{\max}$, as follows.

(4) $$\begin{array}{cc} \max_{W,\;0⪯\vec{p}} & \;\;\;\;\eta =\sum_{i\in N}\eta_{i}^{EE}\\ s.t. & \;\;\;\;p_{i}{\left|g_{i,c}^{H}w_{i}\right|}^{2}\leq I_{\max},\;\;\;\;i\in N\\ \; & \;\;\;\;p_{i}\leq P_{\max},\;\;\;\;i\in N\\ \; & \;\;\;\;∥w_{i}{∥}^{2}=1,\;\;\;\;i\in N, \end{array}$$

where $\vec{p}=\left\{p_{1},p_{2},\cdots ,p_{N}\right\}$, $W=\{w_{1},w_{2},\cdots ,w_{N}\}$, and $P_{\max}$ is the maximum transmit power for each SU Tx. Given that (4) is a non-convex problem due to the co-channel interference, deriving the optimal values of W and $\vec{p}$ mathematically is intractable. Although the optimal solutions can be numerically obtained by an exhaustive search, in which W and $\vec{p}$ are quantized with equally spaced values and all possible combinations are examined in a centralized manner, this requires high computational complexity and message passing overheads for sharing the channel state information (CSI) of all channels.

## 3. Proposed Algorithm

In this section is the proposal of an energy-efficient beamforming and power allocation strategy that can be operated in a distributed manner. In the absence of the knowledge of CSI for interference channels, the optimal beamforming strategy for maximizing the energy efficiency of each SU pair is the maximum ratio transmission (MRT) [15]. Thus, the beamforming vector for SU Tx *i* can be set to $w_{i}=\frac{g_{i,i}}{∥g_{i,i}∥}$.

With $w_{i}=\frac{g_{i,i}}{∥g_{i,i}∥}$, (2) is translated to

(5) $$\begin{array}{cc} r_{i} & =\log_{2}\left(1+\frac{p_{i}{∥g_{i,i}∥}^{2}}{\sigma^{2}+p_{c}|g_{c,i}{|}^{2}+\sum_{j\in N\backslash \{i\}}p_{j}{\left|{\hat{g}}_{j,i}\right|}^{2}}\right), \end{array}$$

where $|{\hat{g}}_{j,i}{|}^{2}=\frac{|g_{j,i}^{H}g_{j,j}{|}^{2}}{∥g_{j,j}{∥}^{2}}$. The interference on the PU Rx caused by SU Tx *i* can also be transformed to    

(6) $$\begin{array}{cc} I_{i} & =p_{i}\frac{|g_{i,c}^{H}g_{i,i}{|}^{2}}{∥g_{i,i}{∥}^{2}}\\ \; & =p_{i}{\left|{\hat{g}}_{i,c}\right|}^{2}. \end{array}$$

With the determined beamforming vector, the optimization problem is reduced to finding the transmit power of Tx *i* that maximizes its own energy efficiency, and this can be developed as follows.

(7) $$\begin{array}{cc} \max_{0\leq p_{i}} & \;\;\;\;\eta_{i}^{EE}\\ s.t. & \;\;\;\;p_{i}{\left|{\hat{g}}_{i,c}\right|}^{2}\leq I_{\max}\\ \; & \;\;\;\;p_{i}\leq P_{\max}. \end{array}$$

Defining $q_{i}=\frac{r_{i}}{P_{i}^{CE}}$, the fractional objective function in (7) can be translated into a subtractive form by using nonlinear fractional programming [20]. Problem (7) can then be reformulated as

(8) $$\begin{array}{cc} \max_{0\leq p_{i}} & \;\;\;\;r_{i}-q_{i}P_{i}^{CE}\\ s.t. & \;\;\;\;p_{i}{\left|{\hat{g}}_{i,c}\right|}^{2}\leq I_{\max}\\ \; & \;\;\;\;p_{i}\leq P_{\max}. \end{array}$$

To solve the problem (8) using dual methods [21], first define the following Lagrangian function of (8).

(9) $$\begin{array}{cc} L(p_{i},\lambda_{i},\mu_{i})\; & =\;r_{i}\;-\;q_{i}P_{i}^{CE}\;+\;\lambda_{i}\;\left(\;I_{\max}\;-\;p_{i}{\left|{\hat{g}}_{i,c}\right|}^{2}\;\right)\;+\;\mu_{i}\;\left(\;P_{\max}\;-\;p_{i}\;\right), \end{array}$$

where $\lambda_{i}$ and $\mu_{i}$ are the Lagrange multipliers for the first and second constraints of (8), respectively, which have non-zero values. $\vec{\lambda }=\left\{\lambda_{1},\lambda_{2},\cdots ,\lambda_{N}\right\}$ and $\vec{\mu }=\left\{\mu_{1},\mu_{2},\cdots ,\mu_{N}\right\}$ are also denoted.

Then, the dual objective is defined as

(10) $$\begin{array}{c} G\left(\lambda_{i},\mu_{i}\right)=\max_{0\leq p_{i}}\;\;L\left(p_{i},\lambda_{i},\mu_{i}\right), \end{array}$$

and the dual problem is written as

(11) $$\begin{array}{c} \min_{0\leq \lambda_{i},\;0\leq \mu_{i}}\;\;G\left(\lambda_{i},\mu_{i}\right). \end{array}$$

According to (10) and (11), $p_{i}$ can be updated to maximize $L(p_{i},\lambda_{i},\mu_{i})$ while $\lambda_{i}$ and $\mu_{i}$ are updated to minimize $G(\lambda_{i},\mu_{i})$ in each SU Tx in an iterative manner.

The Karush–Kuhn–Tucker (KKT) conditions with complementary slackness are represented by

(12) $$\begin{array}{cc} \; & \frac{\partial L}{\partial p_{i}}=\frac{∥g_{i,i}{∥}^{2}}{\ln2\;\left(\;p_{i}∥g_{i,i}{∥}^{2}\;+\;\sigma^{2}\;+\;p_{c}|g_{c,i}{|}^{2}\;+\;\;\;\sum_{j\in N\backslash \{i\}}\;\;p_{j}{\left|{\hat{g}}_{j,i}\right|}^{2}\;\right)}-q_{i}-\lambda_{i}{\left|{\hat{g}}_{i,c}\right|}^{2}-\mu_{i}=0 \end{array}$$

(13) $$\begin{array}{cc} \; & \lambda_{i}\left(I_{\max}-p_{i}{\left|{\hat{g}}_{i,c}\right|}^{2}\right)=0 \end{array}$$

(14) $$\begin{array}{cc} \; & \mu_{i}\left(P_{\max}-p_{i}\right)=0 \end{array}$$

(15) $$\begin{array}{cc} \; & p_{i}\geq 0,\;\;\lambda_{i}\geq 0,\;\;\mu_{i}\geq 0. \end{array}$$

The transmit power that satisfies the KKT conditions is found from (12)–(15), as follows.

(16) $$\begin{array}{c} p_{i}^{*}\;=\;\;{\left[\;\frac{1}{\ln2\;\left(\;q_{i}\;+\;\lambda_{i}{\left|{\hat{g}}_{i,c}\right|}^{2}\;+\;\mu_{i}\;\right)}\;-\;\frac{\Theta_{i}}{∥g_{i,i}{∥}^{2}}\;\right]}^{\;+\;\;}, \end{array}$$

where ${\left[\cdot \right]}^{+}=\max\left(\cdot ,0\right)$ and $\Theta_{i}=\sigma^{2}+p_{c}|g_{c,i}{|}^{2}+\sum_{j\in N\backslash \{i\}}p_{j}{\left|{\hat{g}}_{j,i}\right|}^{2}$. Given that $\Theta_{i}$ is the sum of the noise power and interference power from the PU and the other SU pairs in (16), it can be measured by subtracting the signal power transmitted by SU Tx *i* from the total power received at SU Rx *i*. This indicates that the SU pair *i* can determine its transmit power without knowledge of the individual values of the parameters in $\Theta_{i}$.

In addition, the Lagrange multipliers are updated by the gradient algorithm, as follows.

(17) $$\begin{array}{cc} \lambda_{i} & \leftarrow {\left[\lambda_{i}-\kappa \left(I_{\max}-p_{i}{\left|{\hat{g}}_{i,c}\right|}^{2}\right)\right]}^{+}, \end{array}$$

(18) $$\begin{array}{cc} \mu_{i} & \leftarrow {\left[\mu_{i}-\nu \left(P_{\max}-p_{i}\right)\right]}^{+}, \end{array}$$

where κ and ν are step sizes that are sufficiently small to ensure the convergence of iterations.

The proposed algorithm works as described in Algorithm 1. In particular, SU Txs perform the initialization of the transmit powers and the Lagrange multipliers randomly and then determine the beamforming vectors with MRT. They also calculate the energy efficiencies with the determined transmit powers and beamforming vectors. Next, each SU Tx calculates the transmit power based on (16) and updates the Lagrange multipliers based on (17) and (18) iteratively until the transmit powers for all SU Txs converge to the stationary points.

Then, all SUs update the achievable spectral efficiencies and consumed powers with the converged values of the transmit powers and examine the convergence of energy efficiencies iteratively until convergence is achieved. There is no need to share information among SUs to find $w_{i}$ and $p_{i}$ in the proposed algorithm, thereby, enabling a distributed operation. From the fact that $\epsilon^{-2}$ iterations are required to make the norm of the gradient less than ϵ in the worst case [22], i.e., the number of iterations for the convergence of inner loop, the computational complexity of the proposed algorithm is $O\;\left(I_{c}N^{2}\epsilon^{-2}\right)$, in which O(·) denotes the big-*O* notation, $N^{2}$ is the number of computations for the calculation of $\vec{p}$, and $I_{c}$ is the number of iterations for the convergence of the outer loop [23].
**Algorithm 1** Energy-efficient beamforming and power allocation.1: Initialize ${\vec{p}}^{\left(0\right)}$, $\vec{\lambda }$, and $\vec{\mu }$, randomly2: Determine $w_{i}=\frac{g_{i,i}}{∥g_{i,i}∥}$, ∀i∈N3: **repeat**4:     Set $\vec{q}=\vec{r}/{\vec{P}}^{CE}$5:     j←16:     **repeat**7:         ${\vec{p}}_{\mathrm{old}}\leftarrow {\vec{p}}^{\left(j-1\right)}$8:         **for** i=1 to *N*9:             Compute $p_{i}^{\left(j\right)}$ according to (16)10:             Update $\lambda_{i}$ and $\mu_{i}$ according to (17) and (18)11:         **end for**12:         ${\vec{p}}^{\left(j\right)}=\left\{p_{1}^{\left(j\right)},p_{2}^{\left(j\right)},\cdots ,p_{N}^{\left(j\right)}\right\}$13:         j←j+114:     **until** $∥{\vec{p}}^{\left(j\right)}-{\vec{p}}_{\mathrm{old}}∥<\epsilon$15:     Update $\vec{r}$ and ${\vec{P}}^{CE}$ with $\vec{p}$16: **until**$∥\vec{r}-\vec{q}{\vec{P}}^{CE}∥<\epsilon$

## 4. Performance Evaluation and Discussion

The following system parameters are considered as the default to evaluate the performance of the proposed scheme [24,25,26,27,28]: *N* = 2, *K* = 2, $I_{\max}$ = −50 dBm, $P_{\max}=p_{c}=P_{\mathrm{circuit}}$ = 30 dBm, and $\sigma^{2}=-100$ dBm. The nodes are randomly generated over an area of 35 × 35 m, in which the maximum distance between SU Tx and SU Rx in the same SU pair is set to 15 m. The path-loss and multi-path fading are considered to generate wireless channels. For example, the path-loss exponent and the attenuation at a reference distance of 1 m are determined as 3.6 and −30 dB, respectively, for the path-loss model. In addition, the multi-path fading is generated by an independent and identically distributed (i.i.d.) circularly symmetric complex Gaussian (CSCG) random variable with a zero mean and unit variance. The following five schemes are compared for performance evaluation.

- Optimal scheme: The beamforming vector is determined by the MRT, i.e., $w_{i}=\frac{g_{i,i}}{∥g_{i,i}∥}$, and the optimal $\vec{p}$ is obtained by exhaustive search where all possible combinations over 100 equally spaced values of $\vec{p}$ are examined.
- Proposed scheme: $\vec{p}$ is determined according to Algorithm 1 with $w_{i}=\frac{g_{i,i}}{∥g_{i,i}∥}$.
- Maximum sum rate scheme: $\vec{p}$ is determined to maximize the sum rate with $w_{i}=\frac{g_{i,i}}{∥g_{i,i}∥}$.
- Maximum power scheme: $P_{\max}$ is used for each SU Tx with $w_{i}=\frac{g_{i,i}}{∥g_{i,i}∥}$.
- Random power scheme: Randomly generated $\vec{p}$ is used for each SU Tx with $w_{i}=\frac{g_{i,i}}{∥g_{i,i}∥}$.

**Figure 2:**
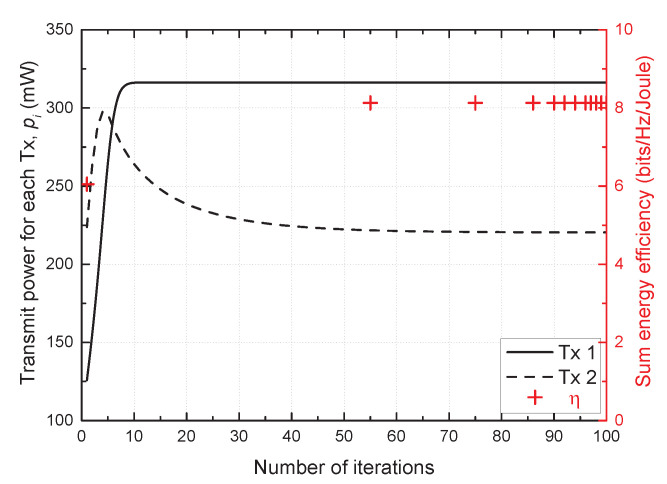
Convergence of the proposed scheme.

As shown in Figure 2, the proposed scheme converged within 60 iterations. More specifically, each SU Tx update transmitted power to maximize its energy efficiency, which, in turn, affected the energy efficiency of the other SU pair. However, the transmit powers of all SU Txs converged as the iteration progressed, and finally the sum energy efficiency also converged. The convergence value of the sum energy efficiency was observed as 8.1, which indicates that the achievable sum rate per unit energy and unit frequency was 8.1 bits.

**Figure 3:**
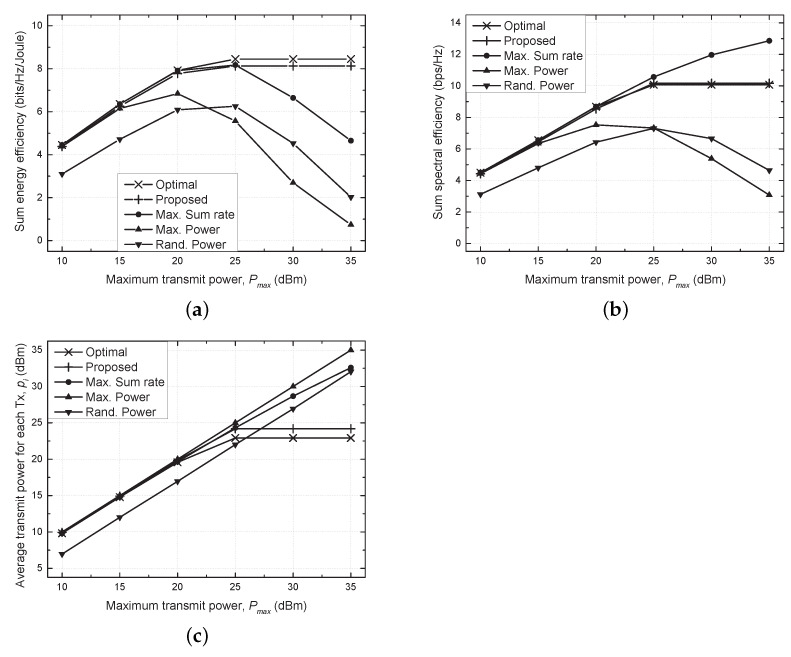
Performance comparison versus the maximum transmit power ($P_{\max}$). (**a**) Sum energy efficiency vs. $P_{\max}$. (**b**) Sum spectral efficiency vs. $P_{\max}$. (**c**) Average transmit power vs. $P_{\max}$.

Figure 3 shows the sum energy efficiency, sum spectral efficiency, and average transmit power versus the maximum transmit power ($P_{\max}$) for all the considered schemes. As the proposed scheme does not use transmit power of more than 25 dBm, which is a loss in terms of energy efficiency, its energy efficiency converges to a stationary point even though $P_{\max}$ increases by more than 25 dBm. The proposed scheme was confirmed to achieve near-optimal performance.

On the other hand, the maximum sum rate scheme used more transmit power to maximize the sum rate as $P_{\max}$ increased. As a result, the sum spectral efficiency improved with increasing $P_{\max}$, but the energy efficiency degraded rapidly when $P_{\max}$ was larger than 25 dBm due to excessive energy use. Both the sum energy and spectral efficiencies were decreased in the maximum and random power schemes, which did not perform adaptive resource management, because of the strong interference as $P_{\max}$ increased.

**Figure 4:**
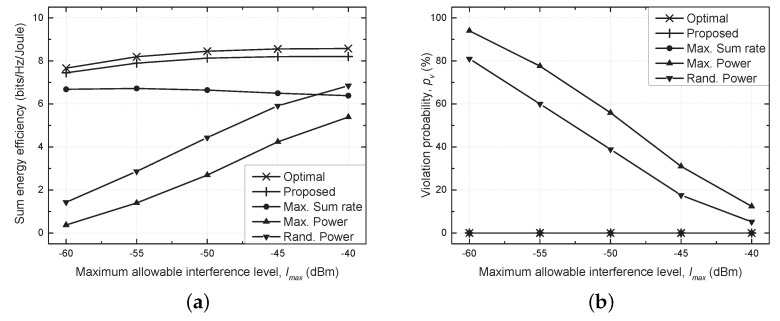
Performance comparison versus the maximum allowable interference level ($I_{\max}$). (**a**) Sum energy efficiency vs. $I_{\max}$. (**b**) Violation probability vs. $I_{\max}$.

Figure 4 depicts the sum energy efficiency and violation probability versus the maximum allowable interference level ($I_{\max}$). Here, the violation probability indicates how much the constraint of allowable interference on the PU Rx is violated, and a penalty is imposed to sum the energy efficiency by setting it to zero if the violation occurs. It is harder to guarantee the constraint of $I_{\max}$ as $I_{\max}$ decreases. As a result, the violation probability increased, and the sum energy efficiency degraded seriously in the maximum and random power schemes, which validates the need for efficient resource management. On the other hand, the violation occurred rarely in the remaining three schemes owing to adaptive resource management.

The proposed scheme performed comparably with the optimal scheme over the entire range of $I_{\max}$, while the maximum sum rate scheme achieved a much lower energy efficiency compared with the proposed scheme because it utilized the power control to maximize the sum rate rather than the sum energy efficiency. When the constraint of the maximum allowable interference was violated, the SU pairs ceased to use the frequency band so as to avoid causing serious interference with the PU Rx. Therefore, a high violation probability limited the use of the frequency band by the SU pairs. Given that the effect of violation is included in the sum energy efficiency by imposing the penalty, the result for the violation probability is omitted in the following results for brevity.

**Figure 5:**
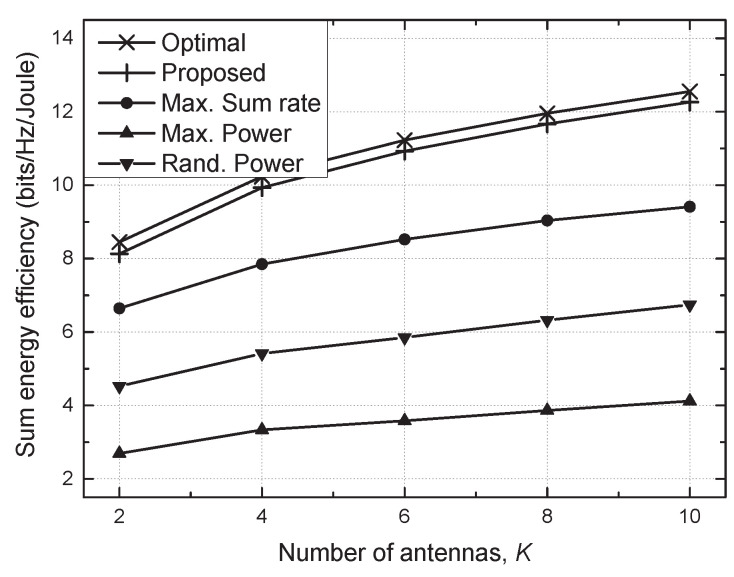
Sum energy efficiency vs. the number of antennas (*K*).

Figure 5 shows the sum energy efficiency versus the number of antennas (*K*). As *K* increased, the sum energy efficiency improved for all considered schemes due to the antenna diversity, which confirms that multiple antennas can generally be utilized to enhance the energy efficiency of the system. The proposed scheme also achieved near-optimal performance for a large-antenna system.

**Figure 6:**
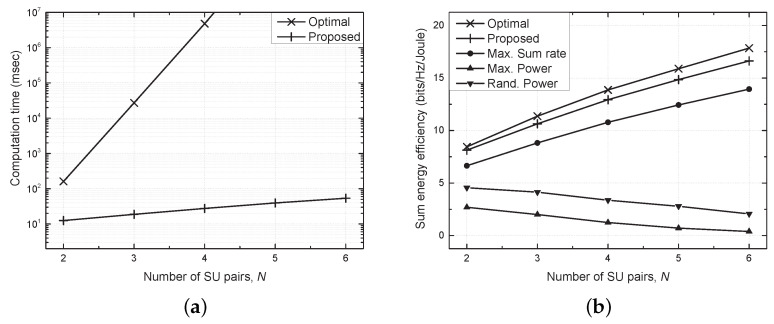
Performance comparison versus the number of SU pairs (*N*). (**a**) Computation time vs. *N*. (**b**) Sum energy efficiency vs. *N*.

Figure 6 shows the performance comparison versus the number of SU pairs (*N*) in terms of the computation time and sum energy efficiency. The proposed scheme achieved a much shorter computation time compared with the optimal scheme, in which the computation time increased exponentially with *N*. As confirmed in Figure 3, SU pairs caused severe interference with each other as *N* increased, which degraded the sum energy efficiency of the maximum and random power schemes. However, the proposed scheme outperformed the conventional schemes by adequately coping with serious interference even for a large number of SU pairs.

## 5. Conclusions

This paper investigated an energy-efficient beamforming and power allocation strategy for CHNs with MISO interference channels, in which the resource allocation for each SU was optimized to maximize its own energy efficiency while guaranteeing the constraint of the allowable interference on primary networks. More specifically, I derived the equations for the beamforming vector and transmit power analytically and proposed an iterative algorithm using dual methods, which is operated in a distributed manner without any information sharing. Our simulation results demonstrated that the proposed scheme not only surpassed the existing ones but also achieved almost optimal performance with a shorter computation time. For future work, our study can be extended to distributed resource management for CHNs with MIMO interference channels.

## Data Availability

Not applicable.
